# Effects of the Uncoupling Protein 1 (UCP1) A-3826G Polymorphism on Taste Preferences in Healthy Young Japanese Adults

**DOI:** 10.3390/life16030499

**Published:** 2026-03-18

**Authors:** Toshishige Kokubun, Tada-aki Kudo, Kanako Tominami, Hirotaka Ishigaki, Ayumu Matsushita, Satoshi Izumi, Takakuni Tanaka, Kotoku Kawaguchi, Yohei Hayashi, Hajime Sato, Naoki Shoji, Keiko Gengyo-Ando, Kazunori Adachi, Junichi Nakai, Guang Hong

**Affiliations:** 1 Division for Globalization Initiative, Tohoku University Graduate School of Dentistry, Sendai 980-8575, Japan; kokubun.toshishige.s6@dc.tohoku.ac.jp (T.K.); ishigaki.hirotaka.t4@dc.tohoku.ac.jp (H.I.); tanatoma@proton.me (T.T.); hong.guang.d6@tohoku.ac.jp (G.H.); 2 Division of Oral Physiology, Tohoku University Graduate School of Dentistry, Sendai 980-8575, Japan; kanako.tominami.c2@tohoku.ac.jp (K.T.); a-matsushita@dent.meikai.ac.jp (A.M.); satoshi.izumi.b8@tohoku.ac.jp (S.I.); keiko.ando.b2@tohoku.ac.jp (K.G.-A.); junichi.nakai.a5@tohoku.ac.jp (J.N.); 3 Division of Pharmacology, Meikai University School of Dentistry, Sakado 350-0283, Japan; k-kawagu@dent.meikai.ac.jp (K.K.); h-sato@dent.meikai.ac.jp (H.S.); k-adachi@dent.meikai.ac.jp (K.A.); 4 Department of Oral Biology, Graduate School of Medical and Dental Sciences, Institute of Science Tokyo, Tokyo 113-8549, Japan; hayashi.y.b6b5@m.isct.ac.jp; 5 Section of Oral-Systemic Health, Oral Science Center, Institute of Biomedical Engineering, Institute of Science Tokyo, Tokyo 113-8549, Japan; 6 School of Food Industrial Sciences, Miyagi University, Sendai 982-0215, Japan; syoujin@myu.ac.jp

**Keywords:** UCP1, single-nucleotide polymorphism (SNP), food preferences, obesity, brown adipose tissue, energy metabolism, high-fat sweet foods

## Abstract

Background: The *UCP1* A-3826G polymorphism, located in the gene’s regulatory region, is associated with obesity and altered fat metabolism. Because UCP1 plays a central role in thermogenesis, variation in its expression may influence metabolic efficiency and dietary fat preference. Methods: We examined associations between the A-3826G polymorphism and food preferences in healthy young Japanese adults (50 males, 48 females). Preferences for high-fat and basic-taste foods were assessed using a self-administered questionnaire, with sweet foods classified as low- or high-fat. Genotypes (AA, AG, GG) were analyzed using a two-way mixed-design ANOVA to evaluate genotype × fat level interactions. Results: Preference scores for basic tastes did not differ significantly among genotypes in either sex (except for sour taste in males). In males, no significant genotype × fat level interaction was observed, although AA carriers preferred high-fat to low-fat sweet foods (*p* < 0.05). In females, a significant genotype × fat level interaction was detected (*p* < 0.01), with AG carriers showing lower preference for high-fat sweet foods. Conclusions: These findings indicate that the UCP1 A-3826G polymorphism may modulate preference for high-fat sweet foods in a sex-dependent manner, suggesting a link between thermogenic genetic variation and dietary fat preference relevant to obesity prevention.

## 1. Introduction

Humans generally prefer foods that contain fat [1,2]. This is partly because foods rich in free fatty acids, such as oleic acid, produce strong reward responses in the mammalian brain [3,4,5]. These fatty acids are reportedly detected by taste cells within the taste buds on the tongue [6,7], suggesting that this mechanism may constitute one of the physiological bases underlying fat preference in human eating behavior. Excessive consumption of high-fat foods, which are typically energy-dense, is a major contributor to obesity [8]. Currently, obesity has become a global epidemic [9,10] and is associated with numerous health problems, including hyperlipidemia and cancer [11,12].

However, eating is a fundamental instinctive behavior essential for maintaining physiological homeostasis, while also providing psychological benefits such as relaxation and satisfaction [13]. Consequently, sustaining long-term calorie restriction is difficult for many individuals. Therefore, identifying factors involved in regulating preferences for high-fat foods—which exert strong influences on appetite and energy balance—is crucial for developing new dietary strategies that are sustainable without excessive effort [14].

Both genetic predisposition and environmental factors contribute to the development of obesity. [10,15]. In particular, polymorphisms in genes related to catecholamine function—especially single-nucleotide polymorphisms (SNPs), the most common form of genetic variation—have been associated with obesity, as catecholamines are key regulators of energy expenditure and lipolysis [16]. Indeed, since adrenergic receptors affect fat metabolism and energy consumption, our research group previously examined the relationship between taste preference and polymorphisms of obesity-related genes: the *β_2_-adrenergic receptor* (*ADRB2*) and *β_3_-adrenergic receptor* (*ADRB3*). Those earlier findings suggested that each of these polymorphisms may be associated with fat preference [14,17].

In mammals, energy expenditure declines during summer, leading to increases in body weight and fat accumulation, which subsequently reduces food intake. Conversely, during winter, energy expenditure rises to maintain body temperature. This seasonal shift occurs through the activation of uncoupling protein 1 (UCP1) in brown adipocytes in response to cold exposure, along with the “browning” of white adipocytes, both of which enhance thermogenesis [18]. As a result, body weight and fat mass decrease, prompting an increase in food intake to compensate for the elevated thermogenic demand. Thus, the *UCP1* gene is thought to play a central role in regulating fat metabolism in mammals by adjusting energy expenditure in accordance with environmental conditions.

Brown adipose tissue (BAT), composed largely of brown adipocytes, serves as the primary site of nonshivering thermogenesis and is essential for both maintaining body temperature and modulating fat accumulation [19,20]. BAT activity diminishes with age and is closely associated with age-related increases in fat deposition [21,22,23,24,25]. UCP1, a mitochondrial protein expressed in BAT, uncouples oxidative phosphorylation from ATP synthesis, thereby dissipating energy as heat to preserve body temperature [19,24,26,27]. Reduced BAT activity has been associated with metabolic disorders such as obesity, insulin resistance, and type 2 diabetes [28,29]. Several lines of evidence have verified the presence of metabolically active BAT in adult humans [21,22,30,31,32], and cold exposure has been shown to activate BAT, contributing to cold-induced thermogenesis [22,23,33,34]. Collectively, these findings indicate that BAT is an important regulator of whole-body energy expenditure and fat accumulation in humans [33].

In BAT, the ADRB3 is the predominant adrenergic receptor subtype, and ADRB3 promotes *UCP1* gene expression and increases thermogenic capacity through the cyclic AMP (cAMP) signaling pathway [24,35,36]. The *UCP1* A-3826G polymorphism has been proposed to be associated with reduced resting energy expenditure as well as diminished cold- or diet-induced thermogenesis [37,38,39,40,41]. Additionally, this variant has been reported to influence body fat accumulation and the efficacy of low-calorie diet therapy in obese individuals [35,42,43]. Nonetheless, the genetic relationship between the *UCP1* A-3826G polymorphism and obesity-related metabolic disorders remains mixed across studies, and the underlying mechanisms have not been fully clarified. For instance, although some studies have shown significant associations between this polymorphism and obesity or body mass index (BMI), others have reported no such association [44,45]. Based on the established role of UCP1 in thermogenesis and fat metabolism, we hypothesized that variation in UCP1 expression associated with the A-3826G polymorphism may influence fat-metabolic efficiency and thereby modulate preference for high-fat foods.

As noted above, the A-3826G polymorphism in the human *UCP1* gene represents a substitution of adenine (A) by guanine (G) in its transcriptional regulatory region, and this change has been suggested to relate to obesity. However, the specific effect of this SNP on food preferences remains uncertain. Therefore, in the present study, we examined the influence of the A-3826G substitution on food preferences in healthy young Japanese adults (mean age: 24.5 years, *n* = 98) using a self-administered questionnaire. Because UCP1 plays an important role in energy metabolism, it may also influence energy intake. Accordingly, to evaluate food preference, we assessed participants’ preferences for four basic taste categories—sweet, salty, sour, and bitter—as well as for high-fat foods.

## 2. Materials and Methods

### 2.1. Study Participants

The participants consisted of healthy young Japanese adults aged 20–39 years who were nonsmokers and not taking any medication. Participants were also required to be in good health at the time of testing. In addition, only individuals whose salivary secretion rates met the following standards were included: at least 1.0 mL/min during gum chewing and at least 0.1 mL/min at rest [17,46]. To reduce the influence of racial or age-related variation on food preferences and health status, we recruited a homogeneous sample of Japanese adults within a narrow age range. Exclusion criteria included smoking and medication use, as both may influence normal physiological functions of the central nervous system, oral functions such as mastication and swallowing, and gustatory perception [47]. Participants whose salivary secretion rates failed to meet the required criteria were also excluded. This criterion was essential because reduced salivation can cause an alteration of taste perception, which directly affects the evaluation of food preference. In addition, reduced salivation may elevate the risk of oral diseases, including dental caries, and contribute to difficulties in mastication, speech, and swallowing [48]. Initially, 119 young adults were recruited through open advertisements, but 21 were excluded because they did not complete all assessments or failed to satisfy the inclusion criteria. Ultimately, data from 98 participants were included in the final analysis.

### 2.2. Study Overview

At the laboratory of the Tohoku University Graduate School of Dentistry, participants completed a self-administered questionnaire that assessed their health status, lifestyle, dietary habits, and taste preferences. They then underwent several examinations, including genotyping of the *UCP1* A-3826G polymorphism, measurement of resting and mastication-induced salivary secretion, and assessment of basic physical characteristics (body weight, height, body fat percentage, and BMI). Body composition was measured using a body composition analyzer (BC-314 or InnerScan 50; Tanita Corporation, Tokyo, Japan) and a custom-built stadiometer.

### 2.3. Genotyping of the UCP1 Gene Polymorphism

To determine the genotype (AA, AG, or GG) of the *UCP1* A-3826G polymorphism, buccal swab samples were obtained from each participant using sterile cotton swabs (Tohmi Co., Ltd., Sakai, Japan). Genotyping was performed by EBS (Hiroshima, Japan) using the previously described procedures [14,17]. Genomic DNA was isolated from buccal samples using the KingFisher Flex purification system (Thermo Fisher Scientific, Waltham, MA, USA) in combination with the MagMAX DNA Multi-Sample Ultra Kit. Genotyping was performed by the polymerase chain reaction with confronting two-pair primers (PCR-CTPP) method using the KAPA2G Robust PCR Kit (Kapa Biosystems, Wilmington, MA, USA) according to the manufacturer’s protocol.

### 2.4. Measurement of Resting and Mastication-Induced Salivary Secretion

Salivary secretion, expressed as a mean salivary flow rate, was assessed for 5 min according to a previously reported protocol [46]. To measure mastication-induced salivary flow, participants were asked to use tasteless and odorless chewing gum (Checkbuf Saliva Test Gum, Horiba, Kyoto, Japan, or an equivalent paraffin gum, Oral Care Co., Tokyo, Japan). During the measurement, participants expectorated accumulated saliva into collection tubes as needed while chewing the gum. They were instructed to abstain from eating or drinking for 2 h prior to testing, remain seated in an upright position throughout the procedure, and rinse their mouths with distilled water before each measurement.

### 2.5. Taste Preference Assessment

To investigate the association between the *UCP1* A-3826G polymorphism and self-reported preferences for the four basic tastes (sweet, salty, sour, and bitter), a self-administered questionnaire was used, based on previously published instruments [14,17,49,50]. Each taste category was evaluated using a five-point scale ranging from 1 (dislike very much) to 5 (like very much). The six sweet food items were divided into low-fat and high-fat groups according to their fat content, as defined by the 2023 Supplement to the Standard Tables of Food Composition in Japan (Eighth Edition) (https://fooddb.mext.go.jp/, accessed on 10 June 2025). Low-fat items (yokan [azuki-bean jelly], manju [steamed bean-jam bun], and candy) contained 0.2–3.3 g fat per 100 g, whereas high-fat items (ice cream, chocolate, and strawberry sponge cake [Japanese-style shortcake]) contained 5.6–40.4 g fat per 100 g. Preference for fatty (high-fat) foods was additionally evaluated using a four-point scale (1–4), consistent with prior studies [14,17,49].

### 2.6. Statistical Methods

Statistical analyses were conducted using JSTAT version 22.1, and Microsoft Excel was used for ancillary data processing. Differences between the two groups were evaluated using unpaired or paired *t*-tests as appropriate, consistent with the comparisons described in each figure. For multi-group or multi-factor comparisons, one-way ANOVA or two-way mixed-design ANOVA was applied as appropriate. As described in the figure legends, when significant effects were detected, Holm’s post hoc test for multiple comparisons was performed to maintain statistical stringency. The chi-square test was used to examine genotype frequency distributions and Hardy–Weinberg equilibrium conformity. For all analyses, a *p*-value < 0.05 was considered statistically significant.

## 3. Results

### 3.1. General Characteristics of the Participants

Table 1 summarizes the general characteristics and data of the study participants. A total of 98 adults (50 males and 48 females; mean age: 24.5 ± 4.5 years; range: 20–39 years) were included in the analysis. The BMI values ranged from 16.0 to 35.4 kg/m^2^, and no significant difference in mean BMI was observed between males and females. With respect to salivary secretion capacity, all participants (*n* = 98) showed normal flow rates, exceeding 0.1 mL/min at rest and 1.0 mL/min during mastication.

### 3.2. Sex Differences in Basic Taste Preference

**Figure 1:**
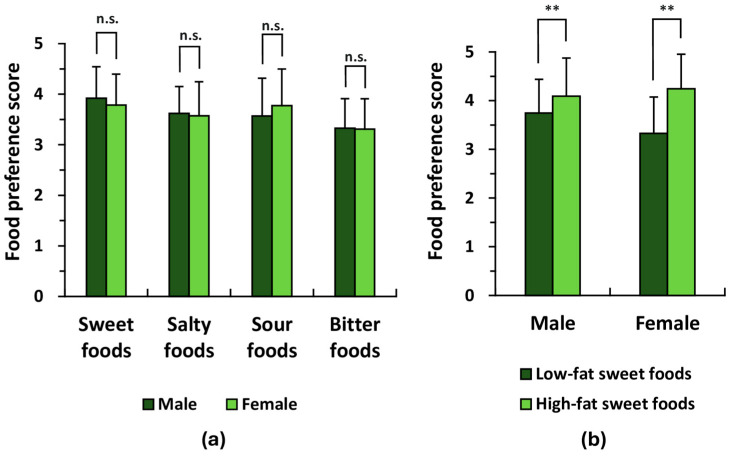
Comparison of basic taste preference scores between male and female subgroups. Preferences for basic tastes were assessed using a self-administered questionnaire. Each participant rated their preference for tastants representing sweet, salty, sour, and bitter tastes on a five-point scale, as listed in Table 2. Participants rated their preferences on a 5-point scale: 5 (like very much), 4 (like moderately), 3 (neither like nor dislike), 2 (dislike moderately), and 1 (dislike very much). (**a**) Comparison of mean taste preference scores across sexes. The aggregated preference scores for each basic taste were evaluated in all participants (*n* = 98). No significant differences were detected between male (*n* = 50) and female (*n* = 48) groups based on the unpaired *t*-test. (**b**) Comparison of mean preferences for higher- and lower-fat sweet food items by sex. In both males and females, significant differences were observed between the mean preference scores for low-fat and high-fat sweet foods. Statistical comparisons were performed using the paired *t*-test. For (**a**,**b**): ** *p* < 0.01; n.s., not significant.

To examine potential sex-related differences in basic taste preference (sweet, salty, sour, and bitter), responses to the questionnaire were analyzed separately for male and female participants. As presented in Table 2, significant differences in preference scores were observed for *yokan* and chocolate, while no notable differences were observed in preference scores for foods such as ice cream, potato chips, lemon, and celery (*p* > 0.05). As shown in Figure 1a, no significant sex-related differences were detected in the mean preference scores for each basic taste (*p* > 0.05). Based on their fat content, the six sweet food items were divided into low- and high-fat groups, and the corresponding mean preference scores were analyzed (see Materials and Methods for details). Notably, both males and females showed a stronger preference for higher-fat sweet items compared with their lower-fat counterparts (*p* < 0.01; Figure 1b).

### 3.3. Genotype Frequencies of the UCP1 A-3826G Polymorphism in Each Sex Group

**Figure 2:**
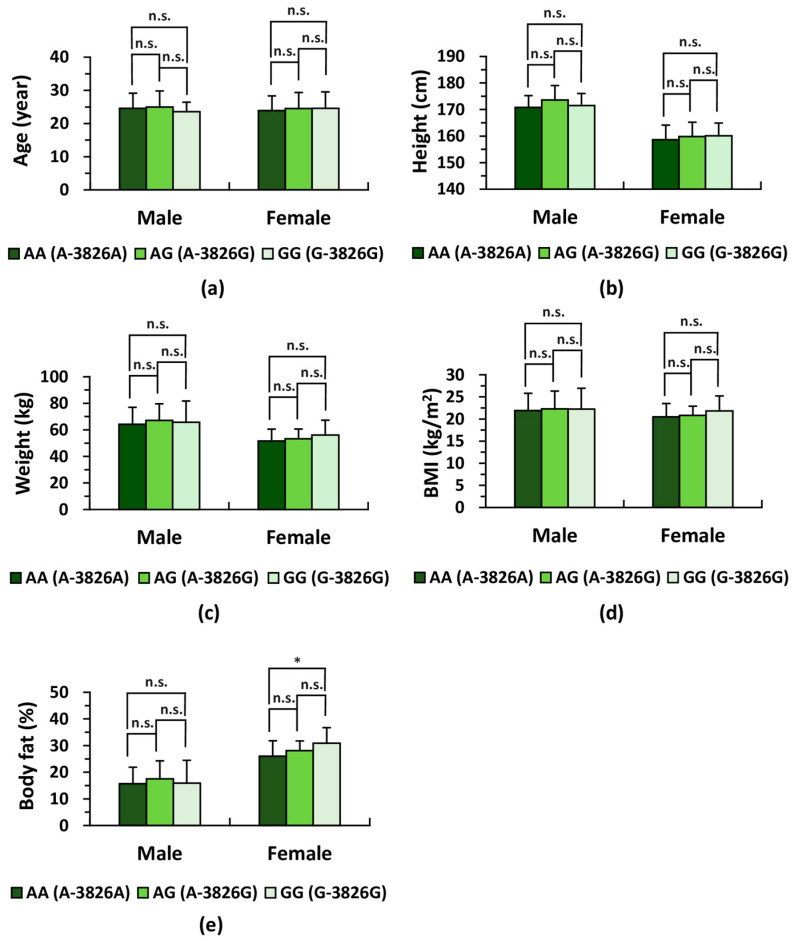
Basic physical characteristics across *UCP1* genotypes within each sex group. The ages of male (*n* = 50) and female (*n* = 48) participants were obtained using a self-administered questionnaire. Basic physical measurements—including body weight, height, body fat percentage (body fat mass/body weight × 100), and BMI—were recorded for all participants (see Materials and Methods for details). (**a**) Comparison of age among *UCP1* genotype subgroups (AA, AG, and GG) in male and female participants. Mean age values were compared using analysis of variance (one-way ANOVA) with Holm’s post hoc test, and no significant differences were detected. (**b**) Comparison of height among *UCP1* genotype subgroups (AA, AG, and GG) in male and female participants. Mean height was compared by one-way ANOVA followed by Holm’s test, revealing no significant subgroup differences. (**c**) Comparison of body weight among *UCP1* genotype subgroups (AA, AG, and GG) in males and females. One-way ANOVA with Holm’s test indicated no significant differences. (**d**) Comparison of BMI among *UCP1* genotype subgroups (AA, AG, and GG) in male and female participants. Mean BMI values were compared using one-way ANOVA and Holm’s test, and no significant differences were observed among the *UCP1* genotype subgroups. (**e**) Comparison of body fat percentage among *UCP1* genotype subgroups (AA and GG) in male and female participants. One-way ANOVA with Holm’s test showed no significant differences in males; however, female participants exhibited a significant difference between the AA and GG subgroups. For (**a**–**e**), * *p* < 0.05; n.s., not significant. BMI, body mass index.

Among the 50 male participants, 18 (36.0%) carried the AA genotype, 23 (46.0%) were AG, and 9 (18.0%) were GG (Table 3). Among the 48 female participants, 12 (25.0%) were AA, 26 (54.2%) were AG, and 10 (20.8%) were GG. In each sex group, the genotype distributions conformed to the Hardy–Weinberg equilibrium, with no significant deviations detected (*p* > 0.05). Each sex group was subsequently divided into the three genotype subgroups (AA, AG, and GG), and their associations with BMI and body fat percentage were evaluated (Figure 2). Although BMI did not differ significantly across genotypes, female individuals with the GG genotype exhibited a significantly higher body fat percentage than those with the AA genotype (*p* < 0.05).

### 3.4. Food Preferences Across UCP1 A-3826G Genotypes Within Each Sex Group

**Figure 3:**
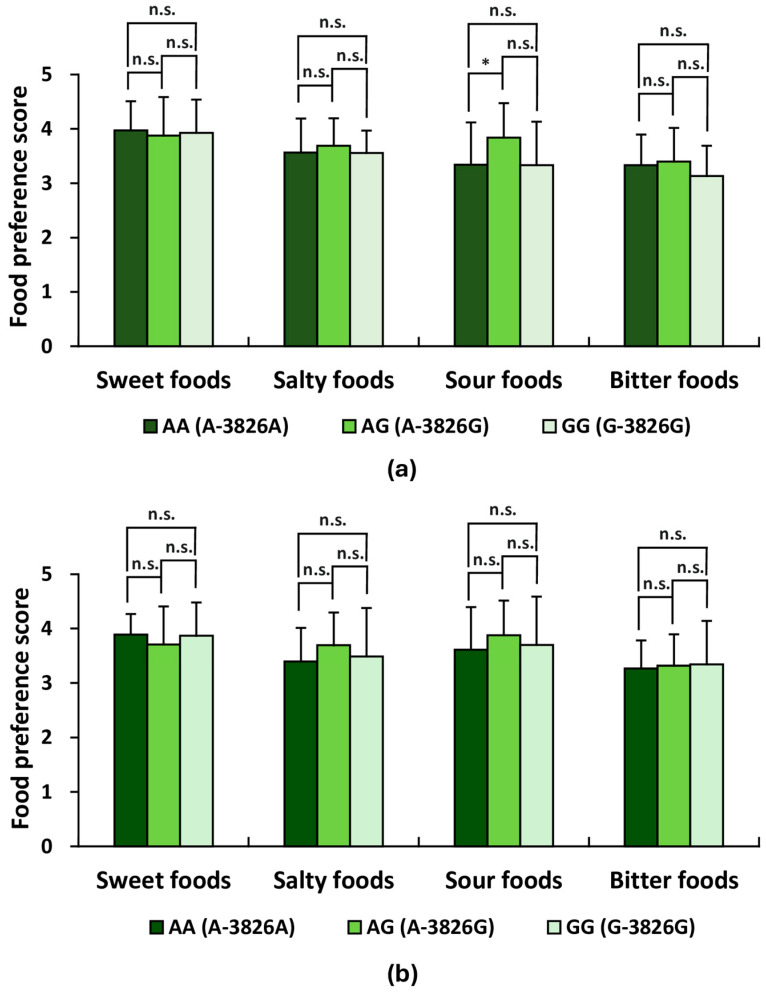
Basic taste preference scores between *UCP1* genotypes within each sex group. Preferences for basic tastes were assessed using a self-administered questionnaire. Each participant rated their preference for tastants representing sweet, salty, sour, and bitter tastes on a five-point scale, as listed in Table 4 and Table 5. Participants rated their preferences on a 5-point scale: 5 (like very much), 4 (like moderately), 3 (neither like nor dislike), 2 (dislike moderately), and 1 (dislike very much). (**a**) Comparison of basic taste preference scores among *UCP1* genotype subgroups (AA, AG, and GG) in male participants (*n* = 50). Mean preference scores for each basic taste were analyzed using ANOVA followed by Holm’s post hoc test. Apart from sour taste, no significant genotype-related differences were detected for any of the basic tastes. (**b**) Comparison of basic taste preference scores among *UCP1* genotype subgroups (AA, AG, and GG) in female participants (*n* = 48). Likewise, in female participants, one-way ANOVA with Holm’s test revealed no significant differences among the genotype subgroups for any of the basic tastes. For (**a**,**b**), * *p* < 0.05; n.s., not significant.

Taste preference scores were also compared across *UCP1* genotype subgroups. Among males, significant differences were observed in preference scores for salted salmon (AG vs. GG, *p* < 0.05) and *miso soup* (AA vs. AG, *p* < 0.01; AG vs. GG, *p* < 0.01). A significant difference was also noted for *mikan* (Japanese orange) (AA vs. AG, *p* < 0.05) (Table 4). Among females, significant differences were observed for chocolate (AG vs. GG, *p* < 0.05; AA vs. AG, *p* < 0.05), pickled vegetables (AA vs. AG, *p* < 0.05), and tea (AA vs. GG, *p* < 0.05; AG vs. GG, *p* < 0.01) (Table 5). Although some individual items differed, aggregated scores for each taste-quality did not show corresponding genotype-related differences. As summarized in Figure 3, male participants displayed significant differences in mean preference scores for sour foods (AA vs. AG, *p* < 0.05), whereas no significant genotype-related differences were found in any of the basic taste preference scores among female participants.

### 3.5. Preferences for Low-Fat and High-Fat Sweet Foods Across UCP1 Genotypes Within Each Sex Group

For the six sweet food items included in the questionnaire, a two-way mixed-design ANOVA revealed a significant interaction between genotype and fat content among female participants (*p* < 0.01), whereas no significant interaction was observed among males.

**Figure 4:**
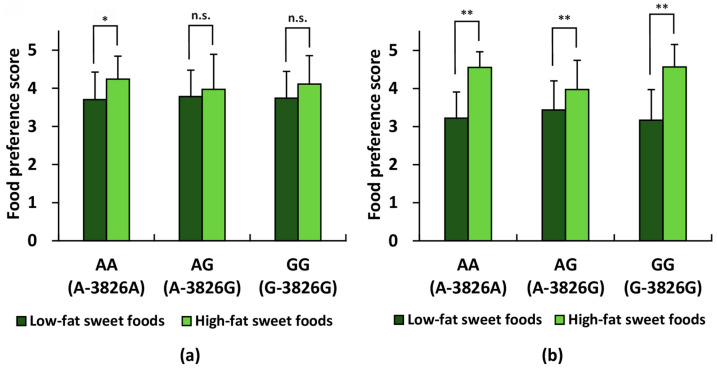
Preference scores for high- and low-fat sweet foods across *UCP1* genotypes within each sex group. Six sweet food items from the self-administered questionnaire were categorized into high-fat (ice cream, chocolate, strawberry sponge cake) and low-fat (*yokan*, *manju*, candy) groups based on fat content. The data were analyzed using a two-way mixed-design ANOVA (genotype × fat level) followed by Holm’s post hoc test. Among males (**a**), the interaction was not significant; however, post hoc comparisons indicated that only the AA genotype group showed significantly higher preference scores for high-fat foods than for low-fat foods. Among females (**b**), a significant interaction was detected, and all genotype groups (AA, AG, GG) exhibited significantly higher preference scores for high-fat foods. For (**a**,**b**): * *p* < 0.05, ** *p* < 0.01 (adjusted *p*-value); n.s., not significant.

Among males, although the overall interaction was not significant, post hoc comparisons indicated that only participants with the *UCP1* AA genotype showed significantly higher mean preference scores for high-fat sweet foods compared with low-fat sweet foods (*p* < 0.05; Figure 4a). This tendency was not observed in the AG or GG subgroups.

In contrast, among female participants, all three genotype groups (AA, AG, and GG) showed significantly higher mean preference scores for high-fat sweet foods compared with low-fat sweet foods (*p* < 0.01; Figure 4b). The magnitude of this difference varied across genotypes, consistent with the significant interaction detected.

### 3.6. Preferences for Fatty Foods Across UCP1 Genotypes Within Each Sex Group

**Figure 5:**
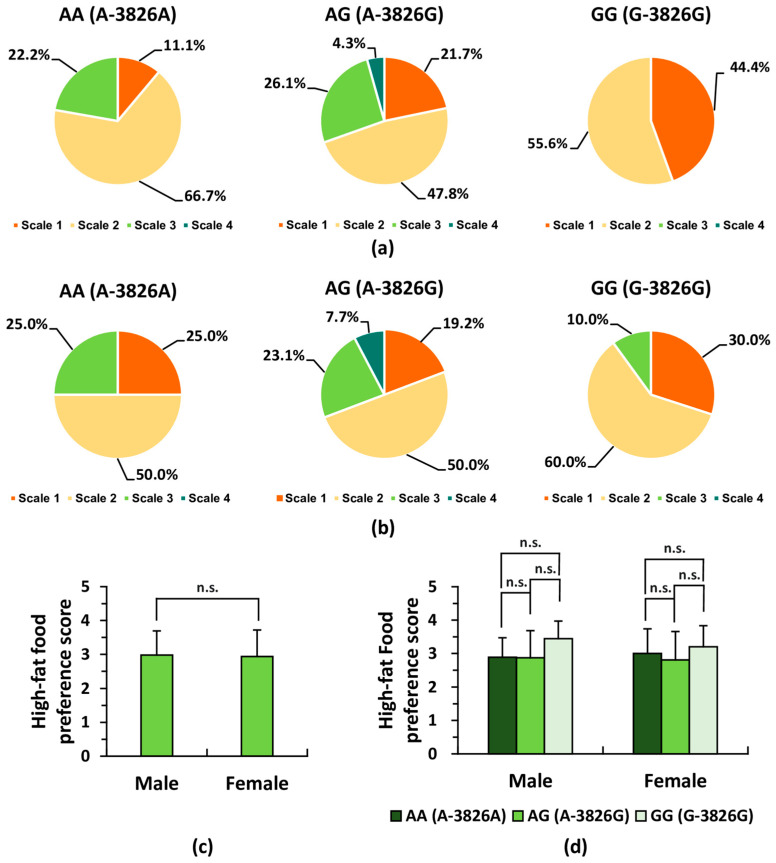
Preferences for fatty foods across *UCP1* genotypes within each sex group. In the self-administered questionnaire, preference for fatty foods was evaluated using a four-point scale, as shown in Table 6: Scale 4 = “dislike very much (score: 1)”; Scale 3 = “dislike moderately (score: 2)”; Scale 2 = “like moderately (score: 3)”; Scale 1 = “like very much (score: 4).” Response distributions (percentage selecting each scale value) and mean preference scores were assessed across genotype groups within each sex. (**a**) Pie chart showing the percentage of male participants selecting each scale value (1–4) within each genotype subgroup (*UCP1* AA: *n* = 18; AG: *n* = 23; GG: *n* = 9; total *n* = 50). No male participants with the GG genotype selected scale 3 or 4. (**b**) Pie chart showing the percentage of female participants selecting each scale value (1–4) within each genotype subgroup (*UCP1* AA: *n* = 12; AG: *n* = 26; GG: *n* = 10; total *n* = 48). No female participants with the GG genotype selected scale 4. (**c**) Comparison of mean preference scores for fatty foods between male and female participants (*n* = 98). Differences between sexes were assessed using an unpaired *t*-test. (**d**) Comparison of mean preference scores for fatty foods among *UCP1* genotype subgroups for each sex subgroup. Statistical significance was evaluated using one-way ANOVA followed by Holm’s post hoc test. For (**c**,**d**); n.s., not significant.

To investigate the association between the *UCP1* A-3826G polymorphism and self-reported preference for fatty (high-fat) foods, specific questionnaire items (Table 6) previously employed in related studies [14,17,49] were evaluated. The proportion of participants selecting each scale value varied across the *UCP1* genotype subgroups. Notably, in each sex group, a higher proportion of individuals with the *UCP1* GG genotype reported that they “liked” or “liked very much” fatty foods compared with those in the other genotype groups (Figure 5a,b). Nevertheless, no significant differences were detected in the mean preference scores for fatty foods between males and females (Figure 5c, *p* > 0.05), nor were significant differences observed among the *UCP1* genotype subgroups within each sex group (Figure 5d, *p* > 0.05).

## 4. Discussion

Among the Japanese participants included in this study, the frequencies of the G allele at position -3826 of the *UCP1* gene were 41.0% in males and 47.9% in females, values comparable to those reported in earlier investigations of Japanese cohorts [51]. Additionally, the genotype frequencies of the *UCP1* A-3826G polymorphism in each sex group were in Hardy–Weinberg equilibrium, suggesting no apparent deviation from the expected genotype distribution in this cohort. Based on the established role of UCP1 in thermogenesis and fat metabolism, we hypothesized that variation in UCP1 expression associated with the A-3826G polymorphism may influence the efficiency of fat metabolism and thereby modulate the preference for high-fat foods.

Of the four basic tastes (sweet, salty, sour, and bitter) assessed, males with the *UCP1* AG genotype showed a significantly stronger preference for sour foods than those with the AA genotype, whereas no significant genotype-related differences were detected for the remaining tastes. In females, no significant differences in preference for any of the four tastes were identified among genotypes. However, when sweet foods were subdivided into low-fat and high-fat categories, a two-way mixed-design ANOVA with genotype as a between-subjects factor and dietary fat level (low vs. high) as a within-subjects factor did not detect a significant interaction between genotype and dietary fat level. Post hoc comparisons nevertheless indicated that, among male participants, only the AA genotype subgroup showed a significant preference for high-fat over low-fat sweet foods (*p* < 0.05). While these trends are noteworthy, they should be interpreted cautiously as exploratory results until supported by larger confirmatory studies.

In contrast, among female participants, a significant interaction between genotype and fat content was identified (*p* < 0.01). Although all female genotype groups showed a robust preference for high-fat sweet foods (*p* < 0.01), the AG genotype subgroup exhibited a lower preference compared with the AA and GG subgroups. These findings suggest that while a preference for high-fat sweet foods appears to be widely observed among females, it may be differentially influenced by the *UCP1* polymorphism. Moreover, although mean preference scores for fatty foods did not differ significantly among the AA, AG, and GG subgroups in each sex group, the proportion of individuals who preferred fatty foods tended to be higher in the GG subgroup than in the AA or AG subgroups in both males and females.

The *UCP1* A-3826G polymorphism, recognized as an obesity-related genetic variant, is located at position -3826 upstream of the transcription start site within the *UCP1* promoter. This variant was reported to affect *UCP1* expression and is associated with obesity and related metabolic abnormalities [36,42,52,53,54]. Notably, several studies suggested that *UCP1* expression is lower in obese carriers of the G allele, potentially influencing energy metabolism [51,54]. The A-3826G polymorphism is further associated with weight gain and increased BMI [36,55], and it is regarded as a possible genetic risk factor for obesity [44,56,57]. A 12-year longitudinal analysis also showed that this A-3826G polymorphism is related to increases in body fat [58,59]. In addition, several studies reported an association between the polymorphism and resistance to low-calorie diets as well as various metabolic abnormalities in humans [60,61,62,63,64]. However, to our knowledge, no previous study has examined whether this polymorphism influences basic taste preferences or self-reported preferences for high-fat foods. The present study is the first to explore the relationship between this genetic variation and self-reported food preferences in healthy young Japanese adults.

Our results revealed, for the first time, a significant association between the *UCP1* A-3826G polymorphism and taste preferences in healthy young Japanese adults. The most notable finding was the significant interaction between genotype and dietary fat level observed in female participants, indicating that the influence of *UCP1* variation on preference for high-fat sweet foods is sex-dependent. Although all female genotype groups exhibited a preference for high-fat over low-fat sweet foods, the magnitude of this preference differed according to genotype, suggesting a modulatory effect of the polymorphism. In contrast, among males, only a subgroup-specific pattern was detected, with a greater preference for high-fat sweet foods observed exclusively in individuals with the AA genotype. Additionally, although mean preference scores did not differ significantly among genotypes, descriptive trends suggested that individuals in the GG subgroup were less likely to report a strong dislike of high-fat foods compared with those carrying the AA or AG genotypes. More precisely, among male participants, none of those with the GG genotype rated high-fat foods as “dislike” or “dislike very much,” and among female participants, only 10% of individuals in the GG subgroup selected these ratings. These findings raise the possibility that differences in *UCP1* expression levels within the mitochondria of brown and browned white adipocytes may influence the efficiency of fat metabolism and potentially relate to differences in food preference patterns. However, the mechanisms involved remain unclear.

In this context, previous studies showed that the combination of fat and sugar produced hyperpalatable foods that elicited strong reward responses in the human brain [1,65]. Such foods, unlike those composed of fat alone, may exert distinct effects on neural pathways that regulate human taste preferences. Nonetheless, the precise mechanisms underlying these relationships remain unresolved and warrant further investigation.

Although genotype-related modulation was evident in females, sex hormones or other regulatory factors may influence the magnitude or pattern of these effects relative to those observed in males. The basis of this sex-specific pattern remains an important topic for future research. Additionally, although the male AA subgroup exhibited a stronger preference for high-fat sweet foods than for low-fat sweet foods, descriptive trends indicated that individuals in the GG subgroup of each sex group were less likely to report moderate or strong dislike of high-fat foods compared with other genotype groups. This pattern raises the possibility that reduced UCP1 expression in GG carriers may influence fat metabolism, although the present data do not permit direct conclusions. Future investigations building on these findings will be needed to clarify the mechanisms underlying this tendency.

From a mechanistic perspective, *UCP1* is encoded by the nuclear genome but functions within the inner mitochondrial membrane, where it mediates thermogenesis by uncoupling fatty acid oxidation [19]. Therefore, differences in UCP1 expression associated with the A-3826G polymorphism may directly influence mitochondrial heat production and the efficiency of fat metabolism in brown and beige adipocytes [37,58,66]. Because mitochondrial metabolic status provides feedback to hypothalamic appetite-regulatory circuits [67,68], reduced thermogenic capacity could be associated with an increased preference for energy-dense, high-fat foods. This mechanistic framework may support the biological plausibility of the associations observed in the present study.

Furthermore, recent evidence highlights the role of sex hormones in modulating BAT activity and UCP1 expression. Estrogen has been shown to promote UCP1 expression and enhance thermogenic capacity in brown adipocytes [69,70], potentially buffering genotype-related reductions in UCP1 expression in females. In contrast, testosterone has been reported to suppress UCP1 expression and brown adipocyte activity [71], suggesting that males may lack the hormonal compensation that mitigates the functional impact of the A-3826G polymorphism in females. This may partially contribute to the sex-specific genotype patterns observed in this study.

Although the present study is the first to report an association between the *UCP1* A-3826G polymorphism and fat preference, its precise mechanism remains uncertain. In this regard, UCP1 protein serves as a key regulatory component in fat metabolism, and UCP1 activity influences mitochondrial oxidative metabolism, which is closely linked to the tricarboxylic acid (TCA) cycle [72]. Thus, polymorphism-related differences in UCP1 expression, along with corresponding alterations in adipose tissue fat metabolism, may modulate brain circuits that control feeding behavior, including (i) metabolic homeostasis and (ii) food-reward processing [14,17]. However, the detailed mechanisms involved remain unresolved. Therefore, further research from the perspective of human metabolic regulation is required to determine how variation in the *UCP1* gene influences food preference through brain function. Such studies may help clarify the mechanisms by which the *UCP1* A-3826G polymorphism contributes to fat preference.

In addition, the male-specific increase in sour-taste preference observed in AG males compared with AA males warrants further investigation. At least two considerations warrant discussion: A sour taste often reflects the presence of citric acid, a component of many sour foods such as lemons and oranges. Citric acid is also a central molecule in the TCA cycle, a metabolic pathway that converts carbohydrates, proteins, and fats into water and carbon dioxide to generate energy [73]. Therefore, the increased preference for sour foods observed in AG males may indicate previously unrecognized metabolic changes in energy expenditure associated with the *UCP1* A-3826G polymorphism relative to AA males.

In our investigation of the association between food preference scores and the *UCP1* A-3826G polymorphism, a significant relationship was observed only for miso soup among the seven items classified in the “salty taste” category, and only in male participants. Although the reason for this finding remains unclear, one possible explanation may relate to the characteristic components of miso, the primary ingredient of miso soup. Miso contains relatively high concentrations of kokumi peptides (γ-glutamyl peptides), which are generated during the fermentation process [74]. These peptides contribute to taste richness and continuity [75]. Although small amounts are also present in certain fermented foods such as salted squids (*ika shiokara*) and pickled vegetables (*tsukemono*) [74], miso is known to contain particularly high concentrations. Therefore, the higher preference for miso soup observed in individuals with the AG genotype of *UCP1* may be related, at least in part, to these fermentation-derived peptides. However, the present data do not allow us to determine a causal relationship, and further investigation will be required to clarify this issue.

In the analysis of the association between food preference and *UCP1* genotypes, female G-allele carriers showed a significantly higher preference for tea. Although the underlying reason for this finding cannot be clearly determined in the present study, among the five items included in the “bitter taste” category of our food preference questionnaire, catechins are the bitter compounds that are characteristically abundant in tea [76]. Importantly, the other bitter-taste items in the questionnaire (celery, green pepper, parsley, and coffee) contain negligible amounts of catechins, further highlighting the uniqueness of tea in this regard [77]. Catechins, represented by epigallocatechin gallate, are polyphenolic bitter substances that have been reported to influence not only taste perception but also sympathetic nervous system activity and thermogenesis [78]. Therefore, catechins may be related to the higher tea preference observed in individuals with the AG or GG genotypes of *UCP1*. Nevertheless, further studies are required to clarify this potential relationship, including any causal mechanisms.

Regarding the effects of the *UCP1* A-3826G polymorphism on BMI and body fat percentage, previous studies have reported inconsistent results—some showing significant effects of the A-3826G substitution on BMI and body fat percentage, while others report contradictory findings [45,58,60,62,79,80]. These discrepancies may reflect not only ethnic differences but also variations in age distribution, sex, body composition, lifestyle, and living environments across study cohorts. In the present study of healthy young Japanese adults who were nonsmokers and not taking any medication, no significant associations were found between the A-3826G substitution and BMI. This suggests that although changes in *UCP1* expression and the associated alterations in fat preference (the latter newly demonstrated here to be associated with the *UCP1* A-3826G polymorphism) may influence human body-weight homeostasis, other compensatory mechanisms may counterbalance these effects, thereby preventing detectable differences in BMI.

The precise reason why the A-3826G substitution did not affect BMI remains unclear, but several possibilities can be considered. First, obesity is generally understood to arise not from a single determinant but from the interaction of multiple interrelated factors; therefore, the influence of additional genetic predispositions and environmental conditions cannot be overlooked [81,82]. Second, the participants in the present study were restricted to young adults in their 20s to 30s, a group with a relatively high basal metabolic rate compared with older adults. Indeed, only 4.1% of the participants were classified as obese (BMI ≥ 30). This may have partially compensated for differences in fat preference and metabolic expenditure associated with the A-3826G substitution. Third, the effects of genetic variation on fat preference or fat metabolism may be partially offset by metabolic homeostasis mechanisms—such as appetite regulation—primarily controlled by the hypothalamus.

On the other hand, as noted above, although the *UCP1* A-3826G substitution did not produce significant differences in BMI, it was associated with a significant increase in body fat percentage among female participants. This suggests that while the A-3826G substitution may not exert a sufficiently strong influence to alter BMI, it nonetheless has a measurable effect on fat metabolism in females. Therefore, our findings indicate that the *UCP1* A-3826G substitution may influence not only fat preference but also broader aspects of energy metabolism in young Japanese females. Future research should further examine whether changes in metabolic efficiency drive alterations in fat preference or, alternatively, whether shifts in fat preference occur independently of systemic modifications in fat metabolism.

The present study has several limitations. First, although the relatively small sample size may have limited statistical power, it was comparable to that of previous exploratory studies investigating similar associations. Second, sex differences may influence taste preference. Previous studies have indicated that hormones can affect taste perception and preference [17,83,84]. Consequently, dietary intake and nutrient storage may differ between males and females to meet distinct physiological requirements. Nonetheless, the precise effects of sex on taste preference remain incompletely understood [14,85,86,87]. In the present study, although no significant differences were found in basic taste preferences between male and female participants, genotype analyses were conducted separately by sex in accordance with previous findings. However, the preference scores for each food item represent averaged values, and transient physiological states were not considered. The self-administered questionnaire also did not collect information on hormonal status (including female hormones), and participants were not categorized according to related physiological parameters. Investigating the relationship between hormonal status and taste preference in a sex-specific manner may provide useful insights into the association between the *UCP1* A-3826G polymorphism and taste preference.

Third, in the present study, we did not assess preference for umami, one of the five basic tastes, when evaluating food preferences. This omission reflects the structure of the dietary preference questionnaire employed in the present study, which did not include items specifically designed to evaluate umami taste. We acknowledge this as a limitation of our study and suggest that future research should incorporate umami-specific food items to comprehensively examine the potential associations between the *UCP1* A-3826G polymorphism and taste preferences.

Fourth, because the present study relied on a self-administered questionnaire, individual bias may have influenced the data. Although this approach allowed us to collect large-scale information efficiently, the use of subjective measures limits the precision of taste perception and preference assessments. We acknowledge that the absence of objective taste testing represents a methodological limitation. Future studies should incorporate validated objective taste assessment methods and compare them with subjective ratings to strengthen the interpretation of genotype–phenotype associations. Additionally, integrating physiological markers—such as oral fat sensitivity (e.g., fatty-acid taste thresholds)—may help clarify the multifaceted relationship between *UCP1* variants and dietary behaviors.

Fifth, the questionnaire used in this study assessed preference for each basic taste by presenting several food items that are generally considered to contain a dominant component of that taste and by calculating the mean preference score. This approach is practical and has been widely used; however, real-world foods rarely contain a single dominant taste component in isolation. Most foods include multiple sensory attributes—such as sweetness, sourness, saltiness, umami, fat content, and odor—and their perceived taste varies depending on product formulation and individual experience. For example, foods categorized as salty (e.g., potato chips) also contain considerable amounts of fat and carbohydrates, and some types of *tsukemono,* such as *bettarazuke* are sweet rather than salty. Therefore, the taste categories used in the questionnaire inevitably involve simplification, and the resulting preference scores should be interpreted as practical proxies rather than precise measures of basic taste preference. To achieve a more accurate assessment, future studies should incorporate objective sensory evaluation methods or controlled taste stimuli in addition to questionnaire-based measures.

Finally, the participants in the present study were healthy young Japanese adults who were nonsmokers and not taking any medications. Therefore, caution is warranted when generalizing these findings to broader or more diverse populations.

## 5. Conclusions

This study examined the association between the *UCP1* A-3826G polymorphism and taste preferences in healthy young Japanese adults. The findings indicate a sex-dependent relationship between this polymorphism and preference for high-fat sweet foods, with a significant genotype × dietary fat interaction observed in females. Although the underlying mechanisms remain to be clarified, these results suggest that variation in *UCP1* may contribute to individual differences in fat-related food preferences. By linking thermogenic genetic variation to dietary fat preference, this study provides novel insights into the potential role of energy metabolism–related genes in shaping eating behavior. Such findings may inform future research aimed at elucidating how genetic factors influence dietary behavior and metabolic health.

## Figures and Tables

**Table 1:** Participant Characteristics.

		All			Male			Female			Male vs.
		(Japanese, *n* = 98)			(*n* = 50)			(*n* = 48)			Female
		Mean	SD	Min–Max	Mean	SD	Min–Max	Mean	SD	Min–Max	*p*-Value ^#^
Age (years)		24.5	4.5	20–39	24.6	4.4	20–38	24.4	4.7	20–39	n.s.
Height (cm)		166.0	8.1	151.0–183.5	172.2	5.0	161.5–183.5	159.6	5.2	151.0–175.5	**
Weight (kg)		59.8	12.6	43.3–106.6	65.8	13.1	48.5–106.6	53.5	8.6	43.3–85.7	**
Body mass index (kg/m^2^)		21.6	3.4	16.0–35.4	22.1	4.0	16.0–35.4	21.0	2.6	16.7–30.9	n.s.
Body fat (%)		22.2	8.3	5.6–44.5	16.6	6.8	5.6–34.9	28.2	4.9	16.6–44.5	**

Results are shown as mean ± SD. # Comparisons between male and female participants were performed with an unpaired *t*-test. ** *p* < 0.01; n.s., not significant.

**Table 2:** Food preference in male and female participants.

			All		Male			Female	Male vs.
			(Japanese, *n* = 98)		(*n* = 50)			(*n* = 48)	Female
			Mean	SD	Mean	SD	Mean	SD	*p*-Value ^#^
Sweet food	Azuki-bean jelly (*Yokan*)		3.58	1.09	3.88	1.02	3.27	1.09	**
	Steamed bean-jam bun (*Manju*)		3.87	0.98	4.04	0.86	3.69	1.07	n.s.
	Candy		3.17	1.04	3.32	1.00	3.02	1.06	n.s.
	Ice cream		4.37	0.82	4.34	0.75	4.40	0.89	n.s.
	Chocolate		4.15	0.96	3.96	0.99	4.35	0.89	*
	Strawberry sponge cake (Japanese-style shortcake)		3.98	1.05	3.98	1.08	3.98	1.02	n.s.
Salty food	Potato chips		4.05	0.96	4.04	0.92	4.06	1.00	n.s.
	Salted squids (*Ika shiokara*)		2.72	1.34	2.78	1.27	2.67	1.42	n.s.
	Salted kelp (*Shio kombu*)		3.05	1.20	3.06	1.13	3.04	1.27	n.s.
	Pickled vegetables (*Tsukemono*)		3.44	1.11	3.36	1.06	3.52	1.17	n.s.
	Salted cod roe (*Tarako*)		3.71	1.15	3.82	1.02	3.60	1.27	n.s.
	Salted salmon		3.92	0.88	4.02	0.84	3.81	0.91	n.s.
	Miso soup (*Misoshiru*)		4.29	0.75	4.26	0.75	4.31	0.75	n.s.
Sour food	Japanese orange (*Mikan*)		4.35	0.90	4.38	0.85	4.31	0.95	n.s.
	Hassaku orange		3.66	1.15	3.48	1.11	3.85	1.17	n.s.
	Pickled Japanese plum (*Umeboshi*)		3.24	1.18	3.22	1.11	3.27	1.25	n.s.
	Yogurt		4.02	1.04	3.86	1.14	4.19	0.89	n.s.
	Lemon		3.29	1.07	3.12	1.10	3.46	1.03	n.s.
	Grapefruit		3.46	1.22	3.36	1.22	3.56	1.22	n.s.
Bitter food	Celery		2.41	1.13	2.36	1.03	2.46	1.24	n.s.
	Tea		4.39	0.73	4.36	0.69	4.42	0.77	n.s.
	Green pepper		3.53	1.04	3.52	1.13	3.54	0.94	n.s.
	Parsley		2.57	1.09	2.64	1.10	2.50	1.09	n.s.
	Coffee		3.69	1.25	3.76	1.27	3.63	1.23	n.s.

Results are shown as mean ± SD. # Comparisons between male and female participants were performed with an unpaired *t*-test. ** *p* < 0.01, * *p* < 0.05; n.s., not significant.

**Table 3:** Genotype frequencies of *UCP1* A-3826G polymorphism in male and female participants.

SNP	Male (*n* = 50)					Female (*n* = 48)				
	Ratio of Genotypes			Chi-Square Test		Ratio of Genotypes			Chi-Square Test	
*UCP1*	AA	AG	GG	Χ^2^	*p*-value	AA	AG	GG	Χ^2^	*p*-value
A-3826G	(A-3826A)	(A-3826G)	(G-3826G)			(A-3826A)	(A-3826G)	(G-3826G)		
(refSNP ^#^:	*n* = 18	*n* = 23	*n* = 9	0.121	n.s.	*n* = 12	*n* = 26	*n* = 10	0.349	n.s.
rs1800592)	(36.0)	(46.0)	(18.0)			(25.0)	(54.2)	(20.8)		

*n* indicates the number of participants, and the values in parentheses represent the percentage. The chi-square test was used to assess deviation from the Hardy–Weinberg equilibrium. No significant deviation from the equilibrium was observed for the male and female participants. The *UCP1* A-3826G polymorphism involves a substitution of adenine (A) by guanine (G) and is located at position -3826 upstream of the transcription start site in the promoter region of the *UCP1* gene. n.s., not significant. # RefSNP identification numbers were obtained from public databases where available. SNP denotes single-nucleotide polymorphism.

**Table 4:** Food preferences across *UCP1* A-3826G genotypes in male participants.

				AA		AG		GG				
				(A-3826A)		(A-3826G)		(G-3826G)		AA vs. AG	AA vs. GG	AG vs. GG
				(*n* = 18)		(*n* = 23)		(*n* = 9)				
				Mean	SD	Mean	SD	Mean	SD	*p*-Value ^#^	*p*-Value ^#^	*p*-Value ^#^
Sweet food	Azuki-bean jelly (*Yokan*)			4.00	1.03	3.78	1.09	3.89	0.93	n.s.	n.s.	n.s.
	Steamed bean-jam bun (*Manju*)			3.94	0.80	4.17	0.89	3.89	0.93	n.s.	n.s.	n.s.
	Candy			3.17	0.86	3.39	1.08	3.44	1.13	n.s.	n.s.	n.s.
	Ice cream			4.44	0.62	4.26	0.86	4.33	0.71	n.s.	n.s.	n.s.
	Chocolate			4.22	0.81	3.83	1.07	3.78	1.09	n.s.	n.s.	n.s.
	Strawberry sponge cake (Japanese-style shortcake)			4.06	1.06	3.83	1.15	4.22	0.97	n.s.	n.s.	n.s.
Salty food	Potato chips			3.89	0.90	4.04	1.02	4.33	0.71	n.s.	n.s.	n.s.
	Salted squids (*Ika shiokara*)			2.89	1.13	2.48	1.38	3.33	1.12	n.s.	n.s.	n.s.
	Salted kelp (*Shio kombu*)			3.11	1.18	3.04	1.15	3.00	1.12	n.s.	n.s.	n.s.
	Pickled vegetables (*Tsukemono*)			3.33	1.03	3.43	1.24	3.22	0.67	n.s.	n.s.	n.s.
	Salted cod roe (*Tarako*)			3.72	1.02	3.91	1.12	3.78	0.83	n.s.	n.s.	n.s.
	Salted salmon			4.06	0.87	4.22	0.85	3.44	0.53	n.s.	n.s.	*
	Miso soup (*Misoshiru*)			3.94	0.80	4.70	0.47	3.78	0.67	**	n.s.	**
Sour food	Japanese orange (*Mikan*)			4.06	0.94	4.70	0.70	4.22	0.83	*	n.s.	n.s.
	Hassaku orange			3.11	1.08	3.83	1.11	3.33	1.00	n.s.	n.s.	n.s.
	Pickled Japanese plum (*Umeboshi*)			3.00	1.19	3.39	1.08	3.22	1.09	n.s.	n.s.	n.s.
	Yogurt			3.78	1.11	4.04	1.07	3.56	1.42	n.s.	n.s.	n.s.
	Lemon			3.06	0.94	3.39	1.16	2.56	1.13	n.s.	n.s.	n.s.
	Grapefruit			3.06	1.11	3.70	1.29	3.11	1.17	n.s.	n.s.	n.s.
Bitter food	Celery			2.11	0.76	2.70	1.11	2.00	1.12	n.s.	n.s.	n.s.
	Tea			4.33	0.69	4.43	0.73	4.22	0.67	n.s.	n.s.	n.s.
	Green pepper			3.50	1.15	3.61	1.27	3.33	0.71	n.s.	n.s.	n.s.
	Parsley			2.78	1.06	2.74	1.10	2.11	1.17	n.s.	n.s.	n.s.
	Coffee			3.94	1.11	3.52	1.50	4.00	0.87	n.s.	n.s.	n.s.

Results are shown as mean ± SD. # Differences among *UCP1* genotype groups were evaluated using one-way ANOVA followed by Holm’s post hoc test. ** *p* < 0.01, * *p* < 0.05; n.s., not significant.

**Table 5:** Food preferences across *UCP1* A-3826G genotypes in female participants.

				AA		AG		GG				
				(A-3826A)		(A-3826G)		(G-3826G)		AA vs. AG	AA vs. GG	AG vs. GG
				(*n* = 12)		(*n* = 26)		(*n* = 10)				
				Mean	SD	Mean	SD	Mean	SD	*p*-Value ^#^	*p*-Value ^#^	*p*-Value ^#^
Sweet food	Azuki-bean jelly (*Yokan*)			3.08	1.08	3.38	1.13	3.20	1.03	n.s.	n.s.	n.s.
	Steamed bean-jam bun (*Manju*)			3.75	1.14	3.69	1.05	3.60	1.17	n.s.	n.s.	n.s.
	Candy			2.83	1.11	3.23	1.11	2.70	0.82	n.s.	n.s.	n.s.
	Ice cream			4.75	0.45	4.15	1.05	4.60	0.70	n.s.	n.s.	n.s.
	Chocolate			4.67	0.49	4.04	1.04	4.80	0.42	*	n.s.	*
	Strawberry sponge cake (Japanese-style shortcake)			4.25	0.75	3.73	1.08	4.30	1.06	n.s.	n.s.	n.s.
Salty food	Potato chips			4.08	0.90	3.96	1.04	4.30	1.06	n.s.	n.s.	n.s.
	Salted squids (*Ika shiokara*)			2.25	1.29	2.88	1.31	2.60	1.84	n.s.	n.s.	n.s.
	Salted kelp (*Shio kombu*)			2.67	0.98	3.31	1.26	2.80	1.55	n.s.	n.s.	n.s.
	Pickled vegetables (*Tsukemono*)			2.83	0.83	3.85	1.12	3.50	1.35	*	n.s.	n.s.
	Salted cod roe (*Tarako*)			3.42	1.51	3.69	1.09	3.60	1.51	n.s.	n.s.	n.s.
	Salted salmon			4.08	1.08	3.73	0.83	3.70	0.95	n.s.	n.s.	n.s.
	Miso soup (*Misoshiru*)			4.42	0.67	4.42	0.70	3.90	0.88	n.s.	n.s.	n.s.
Sour food	Japanese orange (*Mikan*)			4.25	0.97	4.46	0.71	4.00	1.41	n.s.	n.s.	n.s.
	Hassaku orange			3.67	1.23	4.00	1.06	3.70	1.42	n.s.	n.s.	n.s.
	Pickled Japanese plum (*Umeboshi*)			3.17	1.11	3.58	1.21	2.60	1.35	n.s.	n.s.	n.s.
	Yogurt			4.08	1.08	4.15	0.88	4.40	0.70	n.s.	n.s.	n.s.
	Lemon			3.17	1.03	3.54	1.07	3.60	0.97	n.s.	n.s.	n.s.
	Grapefruit			3.33	1.23	3.54	1.24	3.90	1.20	n.s.	n.s.	n.s.
Bitter food	Celery			2.00	1.04	2.54	1.14	2.80	1.62	n.s.	n.s.	n.s.
	Tea			4.50	0.52	4.62	0.64	3.80	1.03	n.s.	*	**
	Green pepper			3.83	1.03	3.42	0.90	3.50	0.97	n.s.	n.s.	n.s.
	Parsley			2.75	1.22	2.38	1.06	2.50	1.08	n.s.	n.s.	n.s.
	Coffee			3.25	1.36	3.62	1.20	4.10	1.10	n.s.	n.s.	n.s.

Results are shown as mean ± SD. # Differences among *UCP1* genotype groups were evaluated using one-way ANOVA followed by Holm’s post hoc test. ** *p* < 0.01, * *p* < 0.05; n.s., not significant.

**Table 6:** Fatty food preference across *UCP1* A-3826G genotypes in male and female participants.

Question: Do You Like Fatty Foods?		Genotype: AA (A-3826A)				Genotype: AG (A-3826G)				Genotype: GG (G-3826G)			
		Male		Female		Male		Female		Male		Female	
		(*n* = 18)		(*n* = 12)		(*n* = 23)		(*n* = 26)		(*n* = 9)		(*n* = 10)	
Scale	Score	*n*	%	*n*	%	*n*	%	*n*	%	*n*	%	*n*	%
1. I like them very much	4	2	11.1%	3	25.0%	5	21.7%	5	19.2%	4	44.4%	3	30.0%
2. I like them moderately	3	12	66.7%	6	50.0%	11	47.8%	13	50.0%	5	55.6%	6	60.0%
3. I dislike them moderately	2	4	22.2%	3	25.0%	6	26.1%	6	23.1%	0	0.0%	1	10.0%
4. I dislike them very much	1	0	0.0%	0	0.0%	1	4.3%	2	7.7%	0	0.0%	0	0.0%

Each participant’s fatty food preference score was calculated based on the corresponding question in the self-administered questionnaire.

## Data Availability

The data acquired and analyzed during this study are included in this article. More information is available from the corresponding author on reasonable request.
